# CDX2 downregulation in mouse mural trophectoderm during peri‐implantation is heteronomous, dependent on the YAP‐TEAD pathway and controlled by estrogen‐induced factors

**DOI:** 10.1002/rmb2.12446

**Published:** 2022-02-27

**Authors:** Daisuke Suzuki, Keitaro Okura, Seina Nagakura, Hidehiko Ogawa

**Affiliations:** ^1^ Department of Bioscience Tokyo University of Agriculture Tokyo Japan; ^2^ Research Fellow of Japan Society for the Promotion of Science Tokyo Japan

**Keywords:** blastocyst, cell differentiation, implantation, mouse, trophoblast

## Abstract

**Purpose:**

To investigate the transition of CDX2 expression patterns in mouse trophectoderm (TE) and its regulatory mechanisms during implantation.

**Methods:**

Mouse E3.5–4.5 blastocysts were used to immunostain CDX2, YAP, TEAD4, and ESRRB. Endogenous estrogen signaling was perturbed by administrating estrogen receptor antagonist ICI 182,780 or ovariectomy followed by administration of progesterone and β‐estradiol to elucidate the relationship between the transition of CDX2 expression patterns and ovarian estrogen‐dependent change in the uterine environment.

**Results:**

CDX2 expression was gradually downregulated in the mural TE from E4.0 *in vivo*, whereas CDX2 downregulation was not observed in blastocysts cultured in KSOM. Fetal bovine serum (FBS) supplementation in KSOM induced CDX2 downregulation independently of blastocyst attachment to dishes. CDX2 downregulation in the mural TE was repressed by administration of ICI 182,780 or by ovariectomy, and administration of β‐estradiol into ovariectomized mice retriggered CDX2 downregulation. Furthermore, *Cdx2* expression in the mural TE might be controlled by the YAP‐TEAD pathway.

**Conclusions:**

CDX2 downregulation was induced heteronomously in the mural TE from E4.0 by uterus‐derived factors, the secretion of which was stimulated by ovarian estrogen.

## 1 INTRODUCTION

Implantation is a specific and essential process in mammalian development. Importantly, pregnancy is established through successful implantation. In human, the conception rate is about 30% per menstrual cycle, and three‐quarters of pregnancy losses are attributed to failure of implantation.^1^ In addition, the low efficiency of implantation success is a major hurdle for infertile patients who use assisted reproductive technology (ART) to become pregnant.^2^ Thus, although an understanding of implantation mechanisms is indispensable for enhancing the fertility of mammals, studies on implantation have been limited due to ethical considerations and to the difficulty of direct observation and manipulation.

In mammals, zygotes repeat cleavages and become compacted embryos, termed morulae, while moving in the oviduct. Morulae develop into blastocysts through blastocoel swelling after reaching the uterus. After hatching from the zona pellucida, blastocysts initiate implantation during a limited period called the implantation window.^3^ This window is regulated by ovary‐derived steroid hormones.^3^, ^4^ Especially, estradiol‐17β induces uterine receptivity for blastocysts directly and blastocyst adhesion to the uterus indirectly via endometrial gland secretions such as leukemia inhibitory factor (LIF) and osteopontin (OPN).^5^, ^6^, ^7^, ^8^, ^9^ In mice, estradiol‐17β levels rise transiently during 3.5 days postcoitum (dpc), and the spike of ovarian estradiol‐17β secretion strictly regulates the timing of implantation.^3^, ^10^ In human and rodents, the processes of implantation are subdivided into three steps: apposition, attachment, and invasion. Once ovarian estradiol‐17β stimulates uterine receptivity and blastocyst activation, mouse blastocysts get close to and contact the uterine luminal epithelium (apposition) around E3.5, and start connecting to the uterine tissue (attachment) from E4.0. After the attachment process is completed by E4.5, the blastocysts start invading (invasion) the uterine tissue from E5.0.^10^, ^11^ Therefore, E3.5, 4.0, and 4.5 could be defined as apposition, attachment, and postattachment periods, respectively, in mice.

A blastocyst is constituted of inner cell mass (ICM) and trophectoderm (TE). In early‐stage blastocysts, ICM includes progenitors of epiblast (EPI) and primitive endoderm (PrE), which are the origin of fetus and yolk sac, respectively, in a random “salt and pepper” pattern. Those progenitors are completely committed to EPI and PrE lineages, and PrE‐committed cells localize along the blastocoel following blastocyst development for the implantation.^12^, ^13^, ^14^, ^15^ TE is subdivided into polar and mural parts, which contact and do not contact the ICM, respectively. In mice, polar TE differentiates into placental cells such as trophoblast giant cells (TGCs), spongiotrophoblasts, and labyrinthine trophoblasts. On the other hand, mural TE differentiates into only TGCs to form a yolk sac with PrE‐derived parietal and visceral endoderm, which is indispensable for exchanging nutrients and endocrine signals between mother and fetus before placental formation.^16^, ^17^, ^18^ In addition, mural TE acquires adhesiveness and invasiveness by transforming cell polarity, motility, and intercellular junctions through its differentiation into TGCs to promote implantation.^17^, ^18^, ^19^, ^20^ Thus, the differentiation of mural TE is essential for initiating and advancing implantation in mice. However, the details about mural TE differentiation remain mostly unknown.

CDX2, a member of the caudal‐related homeobox transcription factor gene family, could be initially detected in morula‐stage embryos and become restricted to outer cells (TE progenitors) by the blastocyst stage.^21^ In these stages, CDX2 contributes to the repression of the pluripotent program and the acquisition of TE cell fate in the outside cells.^22^, ^23^, ^24^ This CDX2 expression in preimplantation embryos is regulated by the Hippo signaling pathway. Hippo signaling is inactivated in the outside cells depending on cell polarity, which enables the downstream transcriptional cofactor YAP to access the nuclei, whereas activated Hippo signaling segregates YAP from nuclei via its phosphorylation in the inside cells, which do not possess polarity. Thus, in the outside cells, nuclear YAP binds to TEAD4 and the YAP/TEAD4 complexes induce *Cdx2* expression.^25^, ^26^, ^27^, ^28^, ^29^ On the other hand, CDX2 is also important for maintaining the stemness of mouse trophoblasts for correct placental formation, and this CDX2 expression is induced by epiblast‐derived FGF4 via the MEK‐ERK signaling pathway.^30^, ^31^, ^32^, ^33^ Especially, we previously reported that the abnormal expression of *Cdx2* after differentiation disrupts the expression of some differentiation marker genes in mouse androgenetic embryo‐derived trophoblast stem cells (AG‐TSCs).^34^ Thus, CDX2 possesses dual functions (specification of TE and maintenance of trophoblast stemness) for the morphogenesis of extraembryonic tissues.

Here, we explored the transition of CDX2 expression patterns in mouse peri‐implantation (E3.5, 4.0, and 4.5) blastocysts and its regulatory mechanisms to gain new insights into mural TE differentiation for implantation. The results of this study suggest that mural TE differentiation is heteronomously induced by uterus‐derived factors secreted depending on estrogen signaling.

## 2 MATERIALS AND METHODS

### 2.1 Collection of mouse blastocysts

All mice were purchased from CLEA Japan and maintained in accordance with the Guidelines for the Care and Use of Laboratory Animals, as specified by the Japanese Association for Laboratory Animal Science and by the Tokyo University of Agriculture (approval number: 2020018). The mice were kept in a 12‐light/12‐dark cycle (8:00–20:00 light). To produce ICR and B6D2F1 background blastocysts, female ICR or C57BL/6N mice (>8 weeks of age) were mated with male ICR or DBA2J mice (>10 weeks of age), respectively. E3.5 (14:00), 4.0 (2:00), and 4.5 (14:00) blastocysts were collected by flushing uteri from female mice that had naturally mated with male mice. Some of the E3.5 blastocysts collected by uterine flushing were cultured in KSOM for 24 h to prepare *in vitro* E4.5 blastocysts. To prepare blastocysts for blastocyst culture experiments, female mice were superovulated by intraperitoneal injection of a pregnant mare's serum gonadotropin (PMSG, 7.5 IU) followed 48 h later by human chorionic gonadotropin (hCG, 5 IU) and then mated with male mice or used in *in vitro* fertilization (IVF). The blastocysts were collected by uterine flushing at E3.5 or by culturing in KSOM after IVF until the embryos developed into expanded blastocysts (about 110 h post‐hCG). All experiments below were conducted with ICR background blastocysts. In immunofluorescence analysis of ESRRB, B6D2F1 background blastocysts were also used to clarify whether or not the results are dependent on the mouse strains.

### 2.2 Culture of mouse blastocysts

The zona pellucida (ZP) of each blastocyst was removed by incubation in drops of acid Tyrode's solution (T1788, Sigma‐Aldrich) or 0.5% Pronase (P8811, Sigma‐Aldrich) diluted in M2 medium, after which the ZP‐removed blastocysts were cultured in KSOM or KSOM + 10% fetal bovine serum (FBS) for 24, 48, or 72 h. For hanging drop culture, the ZP‐removed blastocysts were cultured in 20 µl KSOM or KSOM + 10% FBS drops on lids of 6 cm dishes for 30–36 h. To prevent the media from vaporizing, 6 ml PBS was added to each dish. To inhibit the formation of YAP/TEAD4 complexes, the ZP‐removed expanded blastocysts were cultured in KSOM supplemented with verteporfin (17334, Cayman) at 2.5 μM for 4 or 6 h.

### 2.3 Whole‐mount immunofluorescence of mouse embryos

Embryos were fixed in 4% paraformaldehyde for 30 min and permeabilized in 0.2% Triton X‐100/wash solution (0.1% BSA/0.1% PVA/PBS) for 15 min at room temperature (RT). After nonspecific antigens were blocked in 2% BSA, FBS, or donkey serum/wash solution with 0.1% Tween20 for 2 h at RT, embryos were incubated in blocking solution with primary antibodies overnight at 4℃. Primary antibodies were labeled in blocking solution with Alexa Fluor–conjugated secondary antibodies for 1 h at RT. Embryos were transferred to drops of Vectashield with DAPI (H‐1200, Vector Laboratories) diluted in wash solution (1:10 dilution) covered with paraffin liquid oil (26117–45, Nacalai Tesque) on glass‐bottom dishes (D11530H, Matsunami or P53G‐1.5–14‐C/H, MatTek). To reduce nonspecific reactions, 10% BSA/5% donkey serum/wash solution with 0.1% Tween20 was used as a blocking solution when ESRRB antibody was used. Primary antibodies: mouse anti‐CDX2 monoclonal 1:100 (MU392A‐UC, BioGenex); rabbit anti‐CDX2 monoclonal 1:600 (ab76541, Abcam); rat anti‐E‐cadherin monoclonal 1:100 (sc‐59778, Santa Cruz Biotechnology); rabbit anti‐OCT4 monoclonal 1:100 (2840, CST); goat anti‐GATA4 polyclonal 1:50 (sc‐1237, Santa Cruz Biotechnology); rabbit anti‐YAP monoclonal 1:100 (14074, CST); mouse anti‐TEAD4 monoclonal 1:500 (ab58310, Abcam); and mouse anti‐ESRRB monoclonal 1:100 (PP‐H6705‐00, R&D Systems). Secondary antibodies: Alexa Fluor 488 donkey anti‐mouse IgG (A21202, Invitrogen); Alexa Fluor 594 donkey anti‐rabbit (A21207, Invitrogen); Alexa Fluor 488 goat anti‐rat IgG (A11006, Invitrogen); and Alexa Fluor 594 chicken anti‐goat IgG (A21468, Invitrogen). All secondary antibodies were used at a dilution of 1:500. To assess the relationship between the expression of CDX2 and YAP or CDX2 and ESRRB, double immunostaining with mouse anti‐CDX2 monoclonal antibody and rabbit anti‐YAP monoclonal antibody or rabbit anti‐CDX2 monoclonal antibody and mouse anti‐ESRRB monoclonal antibody was performed.

### 2.4 Confocal microscopy and image analysis

Immunofluorescence images were obtained using a confocal laser scanning microscope (LSM710; Carl Zeiss, Oberkochen, Germany). The images were analyzed with LSM software ZEN 2011 and Fiji software (RRID:SCR_002285, https://fiji.sc/#). ^35^ Single‐plane or Z‐stack images (2 μm intervals) were obtained with Plan Apochromat 20x/0.8, Plan Apochromat 40x/1.4 oil, or Plan Apochromat 63x/1.4 oil objectives. To analyze fluorescence intensity of polar and mural TE, single‐plane images of the mid‐section of the blastocysts that included the ICM and blastocoel were obtained. Manual nuclear segmentation along DAPI fluorescence and quantification of CDX2 fluorescence intensity were performed using ROI Manager in the Fiji software. To compare the fluorescence intensity between polar and mural TE, blastocysts were divided into three equal segments (polar, intermediate, and mural) along the embryonic–abembryonic axis, and the fluorescence intensity in each of the cells included in polar and mural segments was measured. The intermediate segments were excluded from the analysis.

### 2.5 Perturbation of endogenous estrogen signaling

Estrogen receptor antagonist ICI 182,780 (500 μg per mouse, fulvestrant, 14409, Sigma‐Aldrich) diluted in 10% DMSO/40% polyethylene glycol 400 (161–09065, FUJIFILM Wako)/50% PBS was intraperitoneally injected into pregnant ICR mice at 2.5 dpc. For a control group, only the solvent was injected. The blastocysts were collected from the mice at 4.5 dpc. To induce the dormant state of blastocysts, we also performed ovariectomy in the morning at 3.5 dpc and subcutaneously injected progesterone (2 mg per mouse, 160–24511, FUJIFILM Wako) diluted in sesame oil (196–15385, FUJIFILM Wako) daily in the evening for 3 days (4.5–6.5 dpc). To prepare activated blastocysts, β‐estradiol (25 ng per mouse, 050–09081, FUJIFILM Wako) diluted in sesame oil was subcutaneously injected about 2 h later after the last progesterone injection. Dormant or activated blastocysts were collected in the morning at 7.5 dpc by flushing uteri.

### 2.6 Statistical analysis

Results are presented as mean ± S.D. of three or more independent experiments. Statistical analysis was performed with the Student's *t*‐test. *p* values less than 0.01 were considered statistically significant.

## 3 RESULTS

### 3.1 CDX2 expression patterns in peri‐implantation blastocysts

**FIGURE 1:**
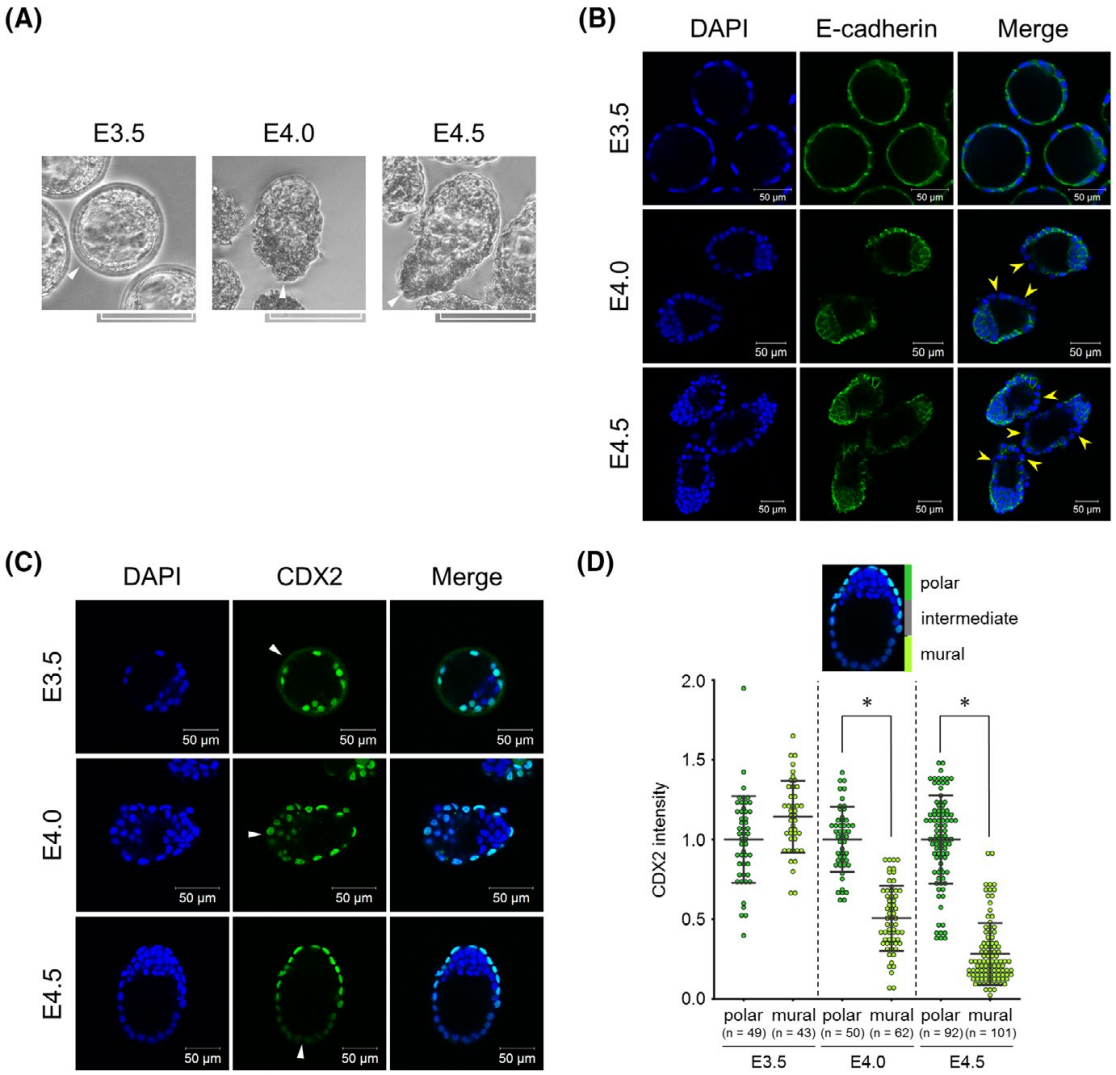
CDX2 expression patterns during implantation. (A) Morphology of E3.5, 4.0, and 4.5 blastocysts collected by flushing uteri. Scale bar: 100 μm. (B) Immunofluorescence analysis of E‐cadherin in E3.5, 4.0, and 4.5 blastocysts. Yellow arrowheads indicate the sites where E‐cadherin localization dissolved. Scale bar: 50 μm. (C) Immunofluorescence analysis of CDX2 in E3.5, 4.0, and 4.5 blastocysts. Arrowheads indicate the tips of mural TE. Scale bar: 50 μm. (D) Comparison of CDX2 fluorescence intensity between polar and mural TE in E3.5, 4.0, and 4.5 blastocysts. Blastocysts were divided into three equal areas (polar, intermediate, and mural) along the embryonic‐abembryonic axis, and the fluorescence intensity in polar and mural areas was measured. Statistical significance was determined by the Student's *t*‐test (*p < 0.01) between polar and mural TE at each stage. n indicates the number of nuclei analyzed. Bars indicate mean ± S.D.

To track the time course of morphology and the CDX2 expression patterns of TE during implantation, we collected E3.5, 4.0, and 4.5 blastocysts by uterine flushing and used them for immunofluorescence of CDX2. E3.5 blastocysts were surrounded by the ZP and showed the well‐known round appearance, while E4.0 and 4.5 blastocysts were hatched from the ZP and apparently became oval‐shaped. Moreover, whereas the E3.5 mural TE had a smooth epithelial surface, E4.0 and 4.5 mural TE gradually became rough (Figure 1A). These results suggested that mural TE started to lose its epithelial property and acquire adhesiveness and invasiveness from E4.0. Actually, the epithelial marker gene E‐cadherin was localized at the basolateral surface at E3.5 but was partially dissolved at E4.0 and E4.5 in the mural TE (Figure 1B). Comparing the fluorescence intensity of CDX2 between polar and mural TE, we found that CDX2 expression in the mural TE gradually decreased from E4.0 and was completely repressed by E4.5, whereas the expression levels were equivalent between polar and mural TE at E3.5 (Figure 1C,D). These results revealed that CDX2 expression was downregulated in the mural TE from E4.0 with implantation progression. We also clarified that mural TE lost the epithelial property in accordance with CDX2 downregulation.

### 3.2 CDX2 expression in mural TE of blastocysts cultured *in vitro*

**FIGURE 2:**
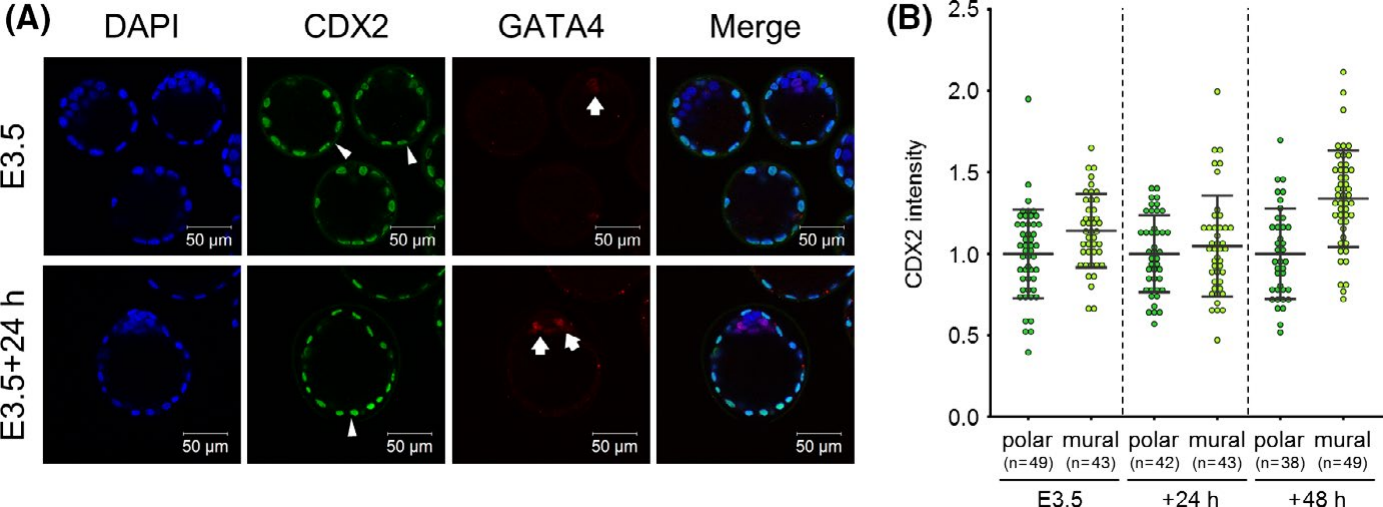
CDX2 expression patterns of *in vitro* cultured blastocysts. (A) Immunofluorescence analysis of CDX2 and GATA4 in E3.5 and *in vitro* cultured (E3.5 + 24 h) blastocysts. Arrows and arrowheads indicate the GATA4‐positive cells and the tips of mural TE, respectively. Scale bar: 50 μm. GATA4 was hardly detected in the ICM of E3.5 blastocysts but was strongly expressed beside the surface of blastocoel cavity in that of E3.5 + 24 h blastocysts. (B) Comparison of CDX2 fluorescence intensity between polar and mural TE in E3.5 and *in vitro* cultured (24 or 48 h) blastocysts. n indicates the number of nuclei analyzed. Bars indicate mean ± S.D.

**FIGURE 3:**
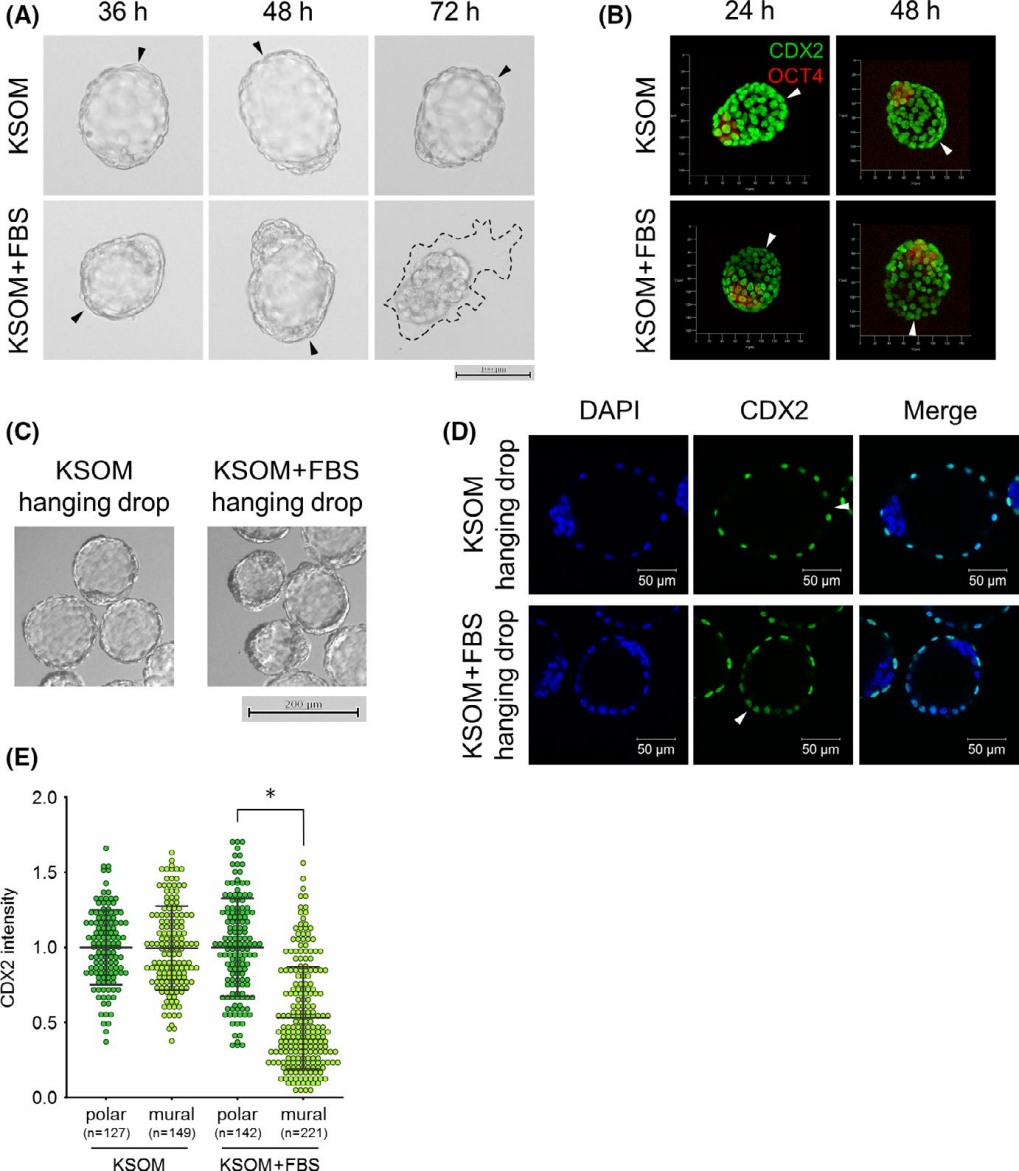
Effect of FBS supplementation on CDX2 expression. (A) Outgrowth development of the blastocysts cultured in KSOM or KSOM supplemented with 10% FBS (KSOM + FBS) for 36, 48, or 72 h. Arrowheads indicate the tips of mural TE. Dashed line indicates the contour of migrating cells (outgrowth). (B) 3D images by maximum intensity projection of CDX2 and OCT4 immunofluorescence in blastocysts cultured in KSOM or KSOM + FBS for 24 or 48 h. Arrowheads indicate the tips of mural TE. (C) Morphology of the blastocysts cultured in hanging drops of KSOM or KSOM + FBS. Arrowheads indicate the thickened mural TE. Scale bar: 200 μm. (D) Immunofluorescence analysis of CDX2 in the blastocysts cultured in hanging drops of KSOM or KSOM + FBS. Arrowheads indicate the tips of mural TE. Scale bar: 50 μm. (E) Comparison of CDX2 fluorescence intensity between polar and mural TE in the blastocysts cultured in hanging drops of KSOM or KSOM + FBS. Statistical significance was determined by the Student's *t*‐test (*p < 0.01) between polar and mural TE in each condition. n indicates the number of nuclei analyzed. Bars indicate mean ± S.D.

Next, we examined whether CDX2 is downregulated in the mural TE of blastocysts cultured *in vitro*. E3.5 blastocysts collected by uterine flushing were cultured in KSOM for 24 or 48 h and used for immunofluorescence of CDX2. To trace PrE specification, PrE marker gene GATA4 was also stained. We selected GATA4 as a PrE marker gene because GATA4 is detected more specifically in PrE‐committed cells.^13^, ^15^, ^36^, ^37^, ^38^ Apparently, blastocysts cultured in KSOM were more expanded than E3.5 blastocysts, and their mural TE maintained a smooth epithelial surface. Immunofluorescence revealed that CDX2 expression was maintained in the mural TE of blastocysts cultured in KSOM (Figure 2A,B). On the other hand, whereas no or only a few cells faintly expressing GATA4 were detected in the ICM of E3.5 blastocysts, GATA4‐positive cells were clearly detected in the ICM of blastocysts cultured in KSOM for 24 h (Figure 2A). These results indicated that CDX2 downregulation in the mural TE is not induced in culture in KSOM, while the differentiation of ICM into epiblast and PrE proceeded autonomously. It has been known that blastocysts start to attach to and spread on a dish (outgrowth) when they are cultured in medium supplemented with FBS, indicating that FBS stimulates the adhesiveness and invasiveness in TE.^39^, ^40^, ^41^, ^42^ In addition, the blastocyst outgrowth assay has been used as an *in vitro* implantation model.^43^ We then cultured E3.5 blastocysts in KSOM with or without 10% FBS for 24, 48, or 72 h after removing ZP, and compared the morphology and CDX2 expression in the mural TE. This revealed that, whereas blastocysts cultured in KSOM maintained an expanded state and never showed outgrowths, blastocysts cultured in KSOM with 10% FBS (KSOM + FBS) developed outgrowths. Moreover, before outgrowth began, cells of mural TE became thick and blastocoel gradually shrank in the blastocysts cultured in KSOM + FBS (Figure 3A). We collected the blastocysts that had attached to but not spread on dishes and subjected them to CDX2 immunofluorescence. Epiblast marker gene OCT4 was also stained to specify the localization of ICM. This revealed that, whereas CDX2 expression was equally detected in polar and mural TE of blastocysts cultured in KSOM, CDX2 was downregulated in the mural TE compared with the polar TE when blastocysts were cultured in KSOM + FBS (Figure 3B). These results prompted us to investigate whether physical contact with the dish is necessary for CDX2 downregulation. Thus, we cultured ZP‐removed E3.5 blastocysts in hanging drops of KSOM or KSOM + FBS. After the culture for 30–36 h, whereas blastocysts cultured in hanging drops of KSOM maintained their expanded state, blastocysts cultured in hanging drops of KSOM + FBS showed thickened mural TE and shrank (Figure 3C). Immunofluorescence and measurement of fluorescence intensity revealed that CDX2 was significantly downregulated in the mural TE of blastocysts cultured in KSOM + FBS hanging drops but not in KSOM hanging drops (Figure 3D,E). Together, these results indicated that some kind of component in FBS induces CDX2 downregulation in the mural TE independently of physical contact *in vitro*.

### 3.3 Effects of inhibiting ovarian estrogen signaling to CDX2 downregulation in mural TE

**FIGURE 4:**
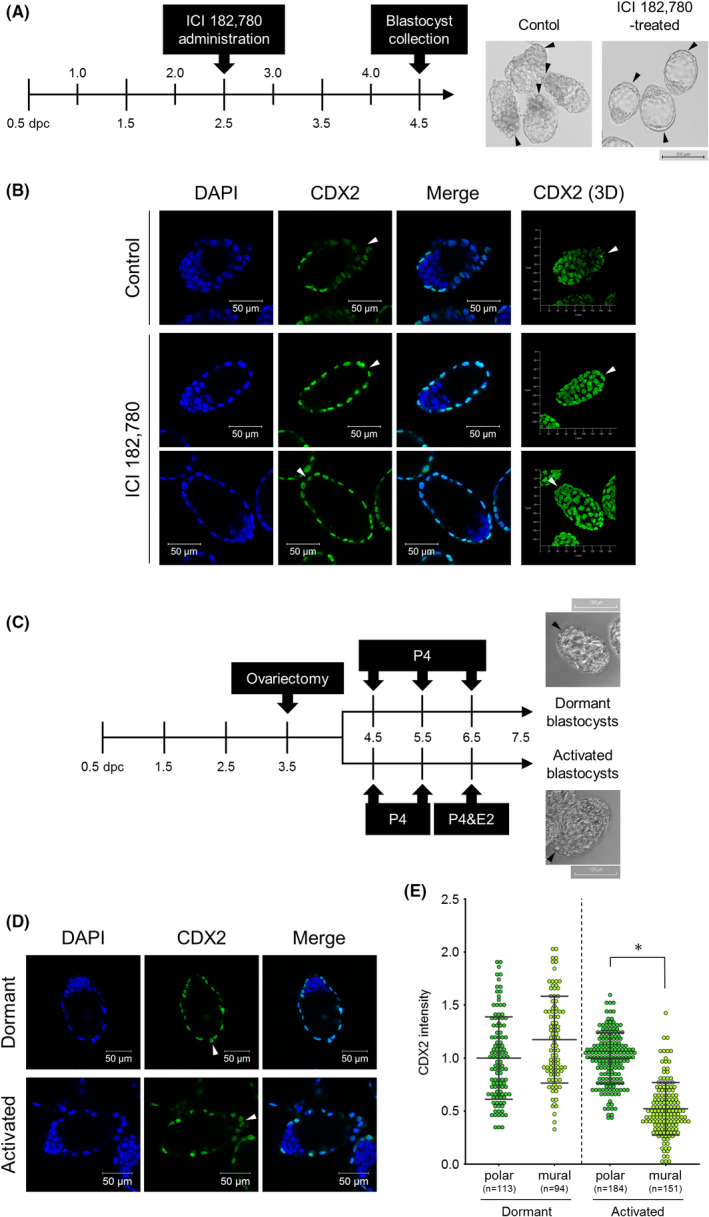
Effect of ovary‐derived estrogen on CDX2 expression. (A) Time schedule of ICI 182,780 injection and blastocyst collection. Pregnant mice were injected with ICI 182,780 at 2.5 dpc, and the blastocysts were collected from them at 4.5 dpc by flushing the uteri. Arrowheads indicate the tips of mural TE. Scale bar: 100 μm. (B) Immunofluorescence analysis of CDX2 in the blastocysts collected from control or ICI 182,780‐treated mice. Arrowheads indicate the tips of mural TE. Scale bar: 50 μm. (C) Time schedule to produce the dormant and activated blastocysts. Arrowheads indicate the tips of mural TE. Scale bar: 100 μm. (D) Immunofluorescence analysis of CDX2 in dormant and activated blastocysts. Arrowheads indicate the tips of mural TE. Scale bar: 50 μm. (E) Comparison of CDX2 fluorescence intensity between polar and mural TE in dormant and activated blastocysts. Statistical significance was determined by the Student's *t*‐test (*p < 0.01) between polar and mural TE in each condition. n indicates the number of nuclei analyzed. Bars indicate mean ± S.D.

On the basis of the above results, we thought that the CDX2 downregulation in the mural TE was heteronomously induced by external factors. It has been known that ovarian estrogen signaling causes the secretion of uterus‐derived factors into uterine fluid to induce blastocyst activation to initiate implantation. We thus hypothesized that ovarian estrogen‐dependent uterine secretion is a factor in inducing CDX2 downregulation in the mural TE. Therefore, we examined the effects of inhibiting ovarian estrogen signaling to the CDX2 downregulation in the mural TE. We first intraperitoneally injected estrogen receptor antagonist ICI 182,780 into pregnant mice at 2.5 dpc and collected E4.5 blastocysts from them (Figure 4A). While the mural TE surface of E4.5 blastocysts from mice injected with only solvent was rough like that of nontreated E4.5 blastocysts, the mural TE of E4.5 blastocysts from the ICI 182,780‐treated mice maintained a smooth surface (Figure 4A). These results indicated that ICI 182,780 administration exactly inhibited the initiation of implantation by ovarian estrogen signaling. Immunofluorescence analysis showed that CDX2 downregulation in the mural TE was repressed in ICI 182,780‐treated blastocysts compared with solvent‐treated blastocysts (Figure 4B). Next, we performed ovariectomy in pregnant mice in the morning at 3.5 dpc, subcutaneously injected them with progesterone once a day for 3 days, and collected dormant blastocysts that are not activated to initiate implantation by obstructing ovarian estrogen secretion. We also collected the activated blastocysts by subcutaneous injection of estradiol‐17β immediately after the last progesterone injection (Figure 4C). Apparently, the mural TE of dormant blastocysts had a smooth epithelial surface, whereas the mural TE surface of activated blastocysts became rough (Figure 4C). Immunofluorescence showed that CDX2 expression in the mural TE was maintained in dormant blastocysts but downregulated in activated blastocysts (Figure 4D,E). Together, these results revealed that the induction of CDX2 downregulation in the mural TE depends on ovarian estrogen signaling. Following a previous report showing that mRNA coding estrogen receptors alpha and beta were not expressed in E3.5 and 4.0 blastocysts,^8^ we suggested that uterus‐derived factors, the secretion of which is induced by ovarian estrogen signaling, induced CDX2 downregulation in the mural TE.

### 3.4 Expression of upstream factors for *Cdx2* expression in mural TE during implantation

**FIGURE 5:**
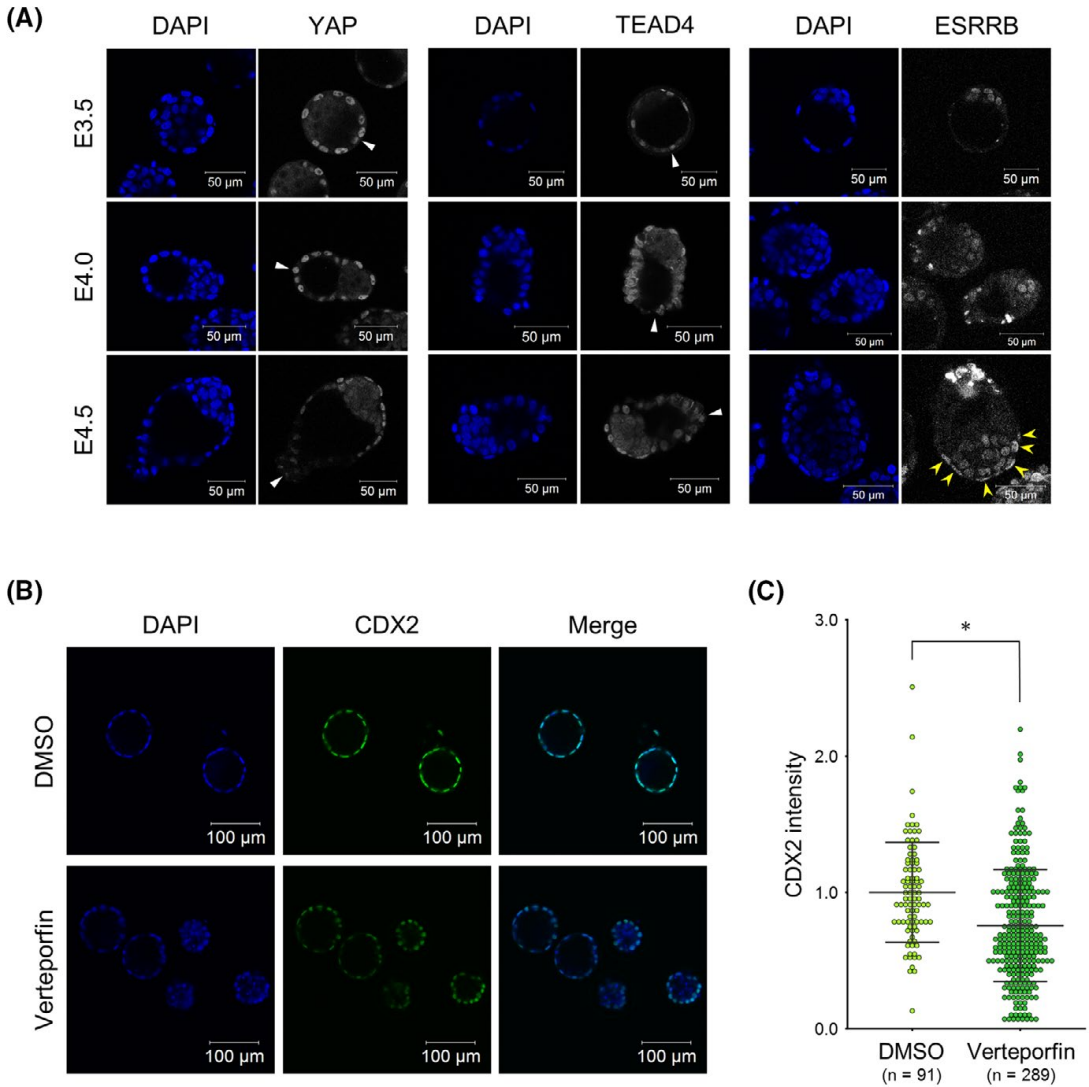
Relationship between CDX2 downregulation and Hippo signaling activity in implantation‐stage blastocysts. (A) Immunofluorescence analysis of YAP, TEAD4, and ESRRB in E3.5, 4.0, and 4.5 blastocysts. White arrowheads indicate the tips of mural TE. Yellow arrowheads indicate the ESRRB‐positive TE. Scale bar: 50 μm. (B) Immunofluorescence analysis of CDX2 in DMSO‐ (control) or verteporfin (VP)‐treated blastocysts. CDX2 expression in TE was found to be weaker in VP‐treated blastocysts than in control blastocysts. Scale bar: 100 μm. (C) Comparison of CDX2 fluorescence intensity in the TE of control and VP‐treated blastocysts. Statistical significance was determined by the Student's *t*‐test (*p < 0.01). n indicates the number of nuclei analyzed. Bars indicate mean ± S.D.

Recently, the question of when regulatory mechanisms upstream of CDX2 expression is switched to MEK‐ERK signaling via epiblast‐derived FGF4 from Hippo‐YAP‐TEAD4 signaling has been discussed. Intriguingly, we detected ESRRB expression only after E4.5 in polar TE, which is regulated by FGF‐MEK‐ERK signaling in mouse trophoblasts (Figure 5A). This result is the same regardless of the mouse strain used (data not shown). This suggested that FGF4‐dependent MEK‐ERK signaling is activated from E4.5 but not from E4.0, while CDX2 downregulation in the mural TE is initiated from E4.0. Therefore, we investigated whether CDX2 downregulation in the mural TE is dependent on Hippo signaling activation. It has been known that Hippo signaling inactivation contributes to the dephosphorylated form of YAP, resulting in its nuclear localization and the formation of YAP‐TEAD4 complexes directly binding and activating the *Cdx2* promoter in preimplantation blastocysts. We then performed immunofluorescence of YAP and TEAD4 and found that both nuclear YAP and TEAD4 in mural TE seemed to be gradually decreased from E3.5 to E4.5 (Figure 5A). In addition, to assess the contribution of YAP/TEAD4 complexes to CDX2 expression in the blastocyst stage, expanded blastocysts prepared by IVF and *in vitro* culture were cultured in KSOM supplemented with verteporfin that inhibits the formation of YAP/TEAD4 complexes for 4 or 6 h. Apparently, whereas control blastocysts remained in the expanded state, verteporfin‐treated blastocysts tended to shrink. CDX2 immunofluorescence showed that CDX2 expression decreased significantly in the TE of verteporfin‐treated blastocysts compared with control blastocysts (Figure 5B,C). These results suggested that the decline of YAP‐TEAD signaling induced CDX2 downregulation in the mural TE and polar TE expressed CDX2 depending on YAP‐TEAD signaling rather than FGF‐MEK‐ERK signaling before E4.5. Together, these results indicated that estrogen signaling‐dependent CDX2 downregulation might be mediated by the decline of YAP‐TEAD signaling.

## 4 DISCUSSION

Previous reports indicated that CDX2 is essential for not only cell fate specification toward TE but also maintaining the stemness of trophoblasts in mice. Thus, in this study, we focused on CDX2 to gain new insights into the differentiation of the mural TE to establish implantation. The present study showed that CDX2 was gradually downregulated in the mural TE during implantation *in vivo*, whereas CDX2 expression was maintained in the mural TE of blastocysts cultured *in vitro*. On the basis of these results, we propose that the differentiation of the mural TE is induced *in vivo* for implantation. In addition, our finding that E4.0 blastocysts already exhibited CDX2 downregulation in the mural TE indicated that mural TE differentiation is initiated at least from E4.0. At this stage, blastocysts attach to the uterine luminal epithelium. Therefore, it was thought that the attachment of blastocysts to the uterine luminal epithelium triggers CDX2 downregulation and mural TE differentiation. However, we also found that CDX2 downregulation was induced by FBS supplementation in KSOM without attachment to a dish *in vitro*. These results suggested that attachment would not be necessary for CDX2 downregulation. Additionally, CDX2‐downregulated mural TE necessarily showed a rough surface not only *in vivo* but also *in vitro*. Furthermore, we found basolateral localization of E‐cadherin was disrupted in mural TE from E4.0. It has been known that *Cdx2* homozygous mutant embryos fail to maintain epithelial integrity by disrupting tight and adherens junctions.^22^, ^44^ Therefore, the mural TE loses its epithelial property via CDX2 downregulation in preparation for implantation.

The initiation of implantation is regulated by ovary‐derived hormones, especially estrogen. It has been known that mouse blastocysts could not initiate implantation and become dormant when estrogen signaling is pharmacologically or physically inactivated. Moreover, a previous report indicated that OPN secreted from endometrial glands depending on estrogen signaling induces the adhesiveness of blastocysts. Thus, ovarian estrogen stimulates blastocysts to initiate implantation indirectly via the secretion of external factors from the uteri. We then examined the relationship between CDX2 downregulation in the mural TE and estrogen signaling, and found that the inactivation of estrogen signaling by the administration of estrogen receptor antagonist or ovariectomy repressed CDX2 downregulation in the mural TE. In addition, administration of estradiol‐17β to ovariectomized mice induced CDX2 downregulation in the mural TE. These findings suggested that the differentiation of the mural TE is also triggered by external factors secreted from uteri depending on estrogen signaling.

FBS includes various ingredients, such as growth factors, hormones, vitamins, lipids, trace elements, and glucose. Remarkably, it has been known that extracellular matrix (ECM) proteins laminin, fibronectin, vitronectin, collagen, and entactin, each possessing the Arg‐Gly‐Asp (RGD) peptide motif, promote blastocyst outgrowth *in vitro* without FBS.^41^, ^45^, ^46^, ^47^, ^48^ In addition, a previous report indicated that interaction between the RGD motif and integrins activates blastocyst adhesion competence by increasing adhesion complex assembly.^8^ The present study revealed that FBS induces CDX2 downregulation in the mural TE, indicating that FBS includes factors that are the same as or analogous to the uterus‐derived factors for not only the activation of blastocyst adhesion competence but also CDX2 downregulation in the mural TE. Further investigation of such factors would be beneficial to understanding the optimal environment for implantation.

Lastly, in this study, we tried to identify the upstream signaling for CDX2 downregulation. It has been known that *Cdx2* expression is induced by Hippo‐YAP‐TEAD4 signaling in preimplantation TE and by FGF4‐MEK‐ERK signaling in postimplantation trophoblasts. Moreover, interestingly, it was revealed that enhancers for *Cdx2* expression differ between blastocysts and TSCs.^49^ Thus, although the regulatory mechanisms of *Cdx2* expression in extraembryonic tissues are well‐known, when and how the regulatory mechanisms and functions of CDX2 switch are still controversial.^50^, ^51^ Recently, Christodoulou et al. suggested that *Cdx2* is expressed depending on FGF4‐MEK‐ERK signaling in E4.75 polar TE.^50^ Additionally, some reports revealed that ESRRB, whose expression is regulated by FGF4‐MEK‐ERK signaling in mouse TSCs, is detected in E4.75 polar TE.^52^, ^53^ In the present study, ESRRB was detected in E4.5 but not in E3.5 and E4.0 polar TE regardless of the mouse strains used, although CDX2 downregulation in the mural TE was started at least from E4.0. Therefore, we proposed here that the onset of CDX2 downregulation in the mural TE is not attributed to switching the regulatory mechanisms of *Cdx2* expression from Hippo‐YAP‐TEAD to FGF‐MEK‐ERK pathway. We also immunostained YAP and TEAD4 and found that both YAP and TEAD4 were downregulated in the mural TE after E4.0, but those expression was maintained in the polar TE, as was the case with CDX2 expression. Moreover, the pharmacological inhibition of YAP‐TEAD4 binding caused CDX2 downregulation in TE. These results suggested that *Cdx2* expression is dependent on Hippo‐YAP‐TEAD signaling before at least E4.5 in TE, and the decline of YAP‐TEAD signaling induces CDX2 downregulation in the mural TE, which contributes to mural TE differentiation.

In conclusion, our results indicated that the differentiation of the mural TE is initiated from peri‐implantation stage E4.0. The results also indicated the possibility that uterus‐derived factors secreted depending on estrogen signaling triggers mural TE differentiation via the decline of YAP‐TEAD signaling. These results suggest the significance of the interaction between embryo and mother for TE differentiation toward implantation. In future research, it will be necessary to identify the uterus‐derived factors that induce TE differentiation, which contributes to efficient pregnancy success by improving the uterus environment.

## CONFLICT OF INTEREST

The authors declare no conflicts of interest.

## HUMAN RIGHTS STATEMENTS AND INFORMED CONSENT

This article does not describe any experiments involving human participants.

## ANIMAL STUDIES

This study was approved by the Ethical Committee for Animal Experiment of Tokyo University of Agriculture. All of the institutional and national guidelines for the care and use of laboratory animals were followed.
